# The Anatomical Variation of the Distal Anterior Cerebral Artery: An Angiographic Study in a Greek Population Sample

**DOI:** 10.7759/cureus.54800

**Published:** 2024-02-24

**Authors:** Christos Chrissicopoulos, Georgios Mavrovounis, Maria Piagkou, George Triantafyllou, Nikolaos Nasis, George Stranjalis, Alexander Andreou, Theodosis Kalamatianos

**Affiliations:** 1 Neurosurgery and Interventional Neuroradiology, Hygeia Hospital, Athens, GRC; 2 Neurosurgery, National and Kapodistrian University of Athens, Athens, GRC; 3 Anatomy, National and Kapodistrian University of Athens, Athens, GRC

**Keywords:** anatomical variations, cerebral vessels, angiography, anterior cerebral artery (aca), callosomarginal artery

## Abstract

Objective

The current retrospective angiographic study establishes the rates of variants in the distal anterior cerebral artery (DACA) in a sample of the Greek population.

Methods

Data were collected from 456 patients who underwent two-dimensional (2D) or three-dimensional (3D) digital subtraction angiography (DSA) of the carotid and vertebral arteries bilaterally. The study focused on patients with good visualization of the anterior and posterior circulations and employed magnetic resonance (MR) or computed tomography (CT) angiography for 3D reconstruction. The anterior cerebral artery (ACA) was classified into one of its two basic configurations, that is, with or without the callosomarginal artery (CMA). The bihemispheric, median, and azygos ACA patterns were also identified.

Results

The majority (373/456, 81.8%) exhibited a typical DACA pattern. The bihemispheric, median, and azygos patterns were identified in 66/456 (14.5%), 10/456 (2.2%), and 7/456 (1.5%), respectively. The CMA was present in 824/912 (90.4%) of the hemispheres, with a trend toward male predominance for bilateral presence (males: 167/192, 86.98%; females: 210/264, 79.55%; p = 0.05). In particular, the CMA was present significantly more frequently (p = 0.002) in the left hemispheres of male patients. Gender differences in CMA presence persisted in the analysis of the patients with a typical DACA pattern.

Conclusion

This study provides insights into the variations of the DACA in the Greek population. The observed gender differences in CMA rates suggest potential morphological variations in cerebral vasculature between males and females and contribute to a better understanding of vascular anatomy for clinical and surgical applications.

## Introduction

The anterior cerebral artery (ACA), one of the two terminal branches of the internal carotid artery, supplies the medial cerebral hemispheres, excluding the medial occipital lobe, and contributes significantly to the anterior blood supply of deep cerebral structures, such as the caudate nucleus, the internal capsule, and the striatum [1]. The ACA is typically divided into five segments (A1-A5), namely, the precommunicating (A1), the infracallosal (A2), the pericallosal (A3), the supracallosal (A4), and the postcallosal (A5) segments [2,3]. Segments A2-A5 are typically termed distal anterior cerebral artery (DACA) [4].

Overall, the mean prevalence of the circle of Willis variants is estimated at 68.2% [5]. Moreover, a large number of angiographic and cadaveric studies have previously investigated the variations in the diameter, course, and branches of the ACA in various populations [4,6,7]. The DACA can be classified into one of two basic configurations, one incorporating and one lacking the callosomarginal artery (CMA) [8,9]. In addition, three major variant patterns have been reported in the literature, namely, the azygos, the median, and the bihemispheric ACA [8]. The azygos ACA is characterized by the absence of an anterior communicating artery and the fusion of the two ACAs to produce a single branch providing cerebral arterial supply to both hemispheres [8]. The median ACA is defined as a vessel branching from the anterior communicating artery, in parallel with the A2 portion of the ACA, which perfuses the corpus callosum and bilateral cerebral hemispheres [8,10]. This variant pattern is also described as triple/triplicate ACA and has been postulated to indicate the persistence of the median artery of the corpus callosum, a remnant of the embryonic circulation [11]. The bihemispheric ACA pattern can be defined as the one displaying branches that originate distal to the first cortical ACA branch to supply the contralateral hemisphere, irrespective of the presence of a hypoplastic or early terminating A2 segment [8,12].

The CMA is an important cortical branch of the ACA that supplies blood to various areas of the brain. Its anatomy has been extensively studied in both cadaveric and angiographic studies and has been the subject of considerable interest. Some authors refer to the CMA as the main branch of the pericallosal artery [9,13], while others mention that it is, along with the pericallosal artery, one of the two terminal branches of the ACA [6,14]. It can arise from almost any segment of the ACA [14], but its most common origin is the A3 segment [8]. The CMA anatomical definitions vary in pertinent literature. In 1974, Moscow et al. proposed that the CMA is the vessel that courses in or near the cingulate sulcus, giving rise to two or more major cortical branches [15]. However, a more recent interpretation by Cavalcanti et al. [9] suggested that the CMA is the vessel that fulfills the definition by Moscow et al. [15] and has the longest pathway in or near the cingulate sulcus.

It is imperative to delineate variants in ACA anatomy, since a clear understanding of the anatomy is crucial for accurate clinical interpretations and surgical interventions involving the anterior cerebral circulation. Thus, the aim of the current study was to explore these variants in a sample of the Greek population via an angiographic approach.

## Materials and methods

Study sample

For the present study, we retrospectively collected data for the patients who underwent two-dimensional (2D) or three-dimensional (3D) digital subtraction angiography (DSA) of the bilateral carotid and vertebral arteries at the Neurosurgery and Interventional Neuroradiology Department of a large tertiary Greek hospital between 2017 and 2020. All studies were performed by a single, experienced interventional radiologist (NN), who interpreted the data together with two experienced neurosurgeons/interventional neuroradiologists (CC and AA). The study was realized in accordance with the principles of the Declaration of Helsinki. All patients provided written informed consent for the use of their personal data, based on Joint Commission International Standards. The protocol of the study was approved by the School of Medicine of the National and Kapodistrian University of Athens (approval number: 45974 {20/10/2020}).

Inclusion and exclusion criteria

The following inclusion criteria were considered in the selection process: (1) patients of all ages and genders (2) who underwent 2D or 3D DSA of the bilateral carotid and vertebral arteries (3) with good visualization of the anterior and posterior circulations; furthermore, (4) the presence of magnetic resonance (MR) or computed tomography (CT) angiography for the 3D reconstruction of the ACAs was also required.

Patients (1) with multiple or giant (>2.5 cm) aneurysms and (2) large (>3 cm) arteriovenous malformations were excluded.

Data collection

The following data were collected for all included patients for both cerebral hemispheres: (1) date of birth, (2) gender, (3) the classification of DACA, and (4) the presence of CMA. The current classification followed Cilliers and Page [8], who defined four different major types of DACA, namely, the typical, the median, the azygos, and the bihemispheric ACA. Regarding the CMA, we implemented the anatomical definition proposed by Cavalcanti et al. [9]. Thus, the CMA was defined as the artery that runs near or inside the cingulate sulcus and gives rise to two or more major branches supplying the cerebral cortex. If more than one artery fulfilled the aforementioned criterion, the CMA was the artery with the longest course near or inside the cingulate sulcus [9].

Statistical analysis

Continuous variables were tested for normality and were presented as medians and interquartile ranges (IQR), while categorical variables were presented as absolute numbers and percentages. The z-score test was used to compare percentages. The chi-squared test with Yates's correction was used to compare proportions between groups. The Mann-Whitney U test or the Kruskal-Wallis H test was implemented to establish differences in ages between groups. The two-tailed statistical significance was set at p < 0.05. All statistical analyses were conducted using the Statistical Package for Social Sciences (SPSS) for Windows version 22.0 (IBM SPSS Statistics, Armonk, NY).

## Results

Study sample characteristics

Overall, 456 patients were included in the present study, with a male-to-female ratio of 192:264 and a median (IQR) age of 52 (39-61) years. The overall sample of DSA studies constituted 912 carotid arteries (456 anteroposterior views and 456 lateral views) and 912 vertebral arteries (456 anteroposterior views and 456 lateral views).

Distal ACA analysis

**Figure 1:**
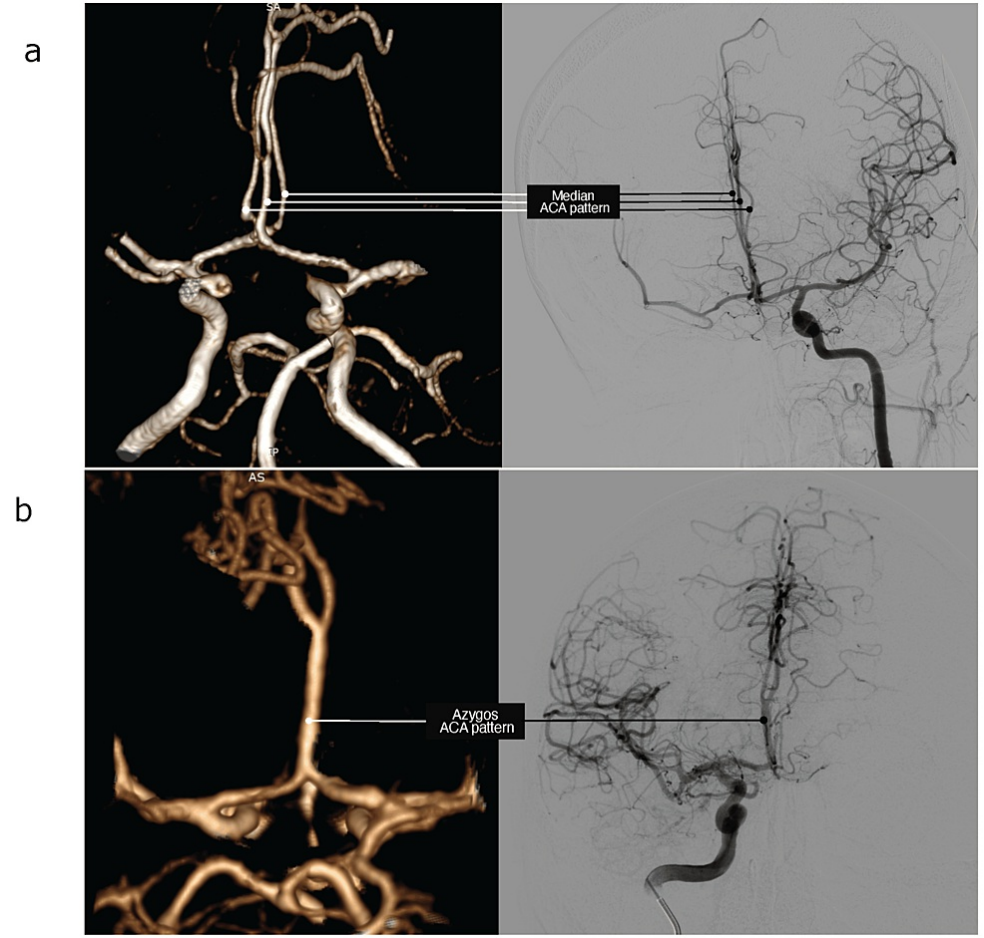
Median and Azygos ACA Angiography images illustrating the (a) median and (b) azygos ACA patterns ACA: anterior cerebral artery

**Figure 2:**
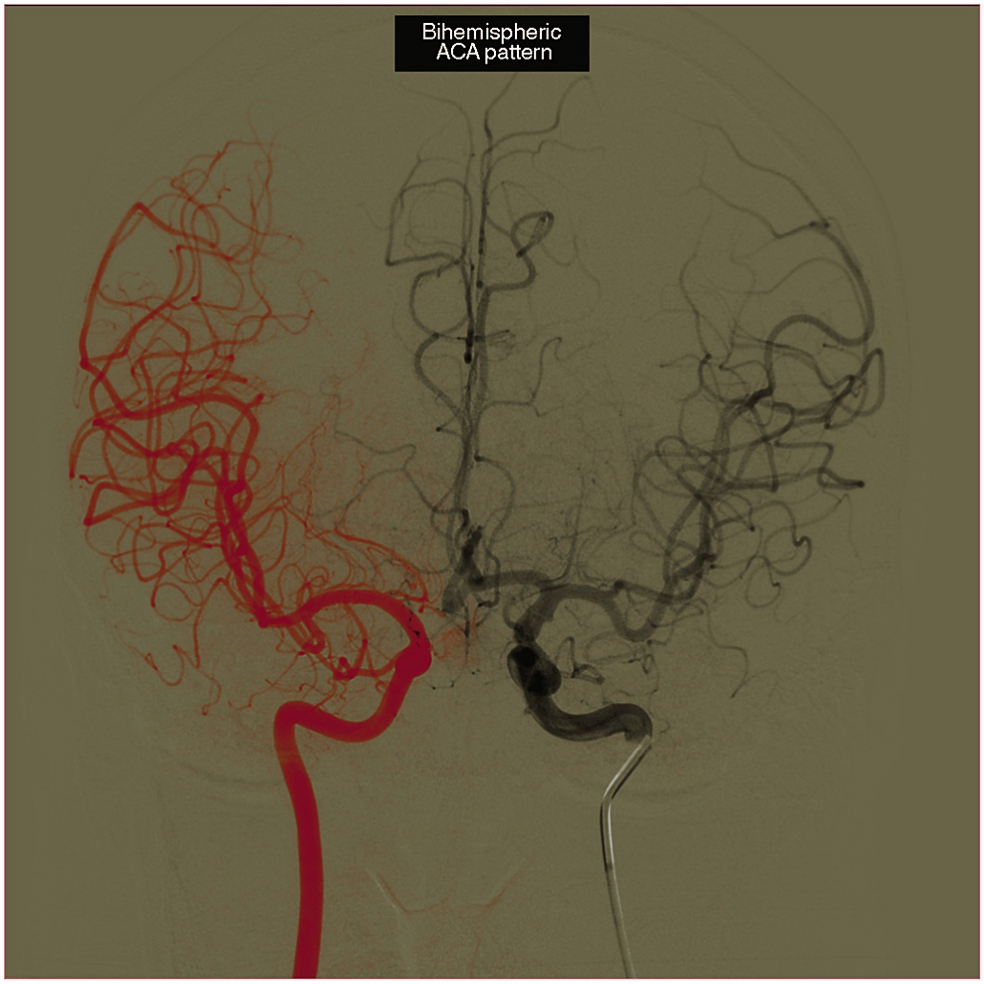
Bihemispheric ACA A collage of two angiography images from the same patient, illustrating the presence of the bihemispheric ACA pattern. Vessels in red, essentially the middle cerebral artery and its branches, are those highlighted after the injection of contrast agent into the right internal carotid artery. Vessels in grey are those highlighted after the injection of contrast agent into the left internal carotid artery. Note the vascularization of frontal regions bilaterally from vessels originating from the left internal carotid artery ACA: anterior cerebral artery

Out of the 456 included patients, 373 (81.8%) had typical DACAs, 66 (14.5%) had a bihemispheric ACA, 10 (2.2%) had a median ACA, and seven (1.5%) had an azygos ACA. Figure 1 and Figure 2 show representative angiographic images of the median, azygos, and bihemispheric ACA patterns.

Out of the 192 included male patients, 153 (79.69%) had typical DACAs, 32 (16.67%) had a bihemispheric ACA, four (2.08%) had a median ACA, and three (1.56%) had an azygos ACA. Out of the 264 included female patients, 220 (83.33%) had typical DACAs, 34 (12.88%) had a bihemispheric ACA, six (2.27%) had a median ACA, and four (1.52%) had an azygos ACA. No statistically significant difference in the median age of patients was identified between the different DACA patterns (p = 0.78).

Callosomarginal artery analysis

Descriptive Characteristics

**Figure 3:**
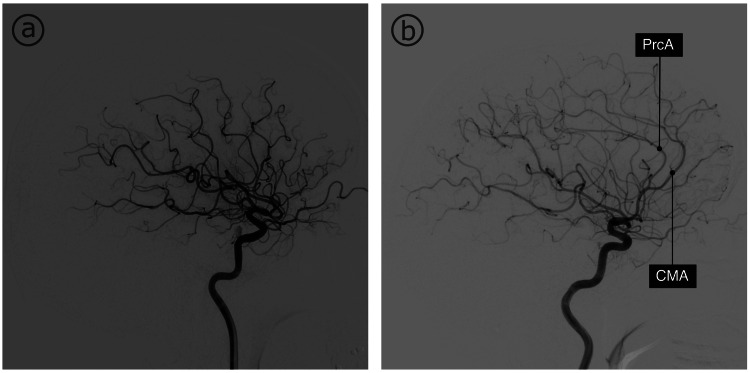
Callosomarginal Artery Angiography images of two patients: (a) one without a CMA and (b) one with a CMA CMA, callosomarginal artery; PrcA, pericallosal artery

Figure 3 shows representative images of the two main ACA configurations, namely, one with and one without the CMA. The CMA was bilaterally present in 377 (82.7%) out of 456 patients, while bilateral absence was identified in nine (2%) of 456 patients. The CMA was present in at least one of the hemispheres in 447 (98%) out of 456 patients. When considering all hemispheres individually, 824 (90.4%) out of 912 patients had a CMA.

Analysis of All Cerebral Hemispheres

The bilateral presence of CMA showed a trend (chi-squared = 3.79; p = 0.05) toward being more common in males (167/192, 86.98%) than in females (210/264, 79.55%). The comparison for the presence of a CMA in at least one cerebral hemisphere did not reach statistical significance (chi-squared = 2.44; p = 0.12), albeit being slightly more common in males (191/192, 99.48%) than in females (256/264, 96.97%).

Regarding lateralization, the CMA was present in 408/456 (89.50%) right hemispheres and in 416/456 (91.20%) left hemispheres (z = 0.90; p = 0.37). No statistically significant difference was identified between the median age of patients with and without a CMA in both the right (p = 0.40) and left (p = 0.77) hemispheres. When a gender-based analysis was conducted, only in the left hemisphere was the CMA more commonly present (chi-squared = 9.81; p = 0.002) in males (185/192, 96.4%) than in females (231/264, 87.5%). For the right hemisphere, the corresponding values were not significant (chi-squared = 0.05; p = 0.83).

Analysis of Cerebral Hemispheres With a Typical Distal ACA Pattern

Α subgroup analysis using only patients with typical DACA was also performed. The bilateral presence of CMA was more common in males (136/153, 88.89%) than in females (181/220, 82.27%), albeit not statistically significant (chi-squared = 2.60; p = 0.11). The comparison for the presence of a CMA in at least one cerebral hemisphere showed a trend (chi-squared = 3.38; p = 0.07) of CMA being more common in males (153/153, 100%) than in females (213/220, 96.82%).

Similar lateralization results were also present. Specifically, the left CMA was more commonly present (chi-squared = 5.87; p = 0.02) in males (149/153, 97.4%) than in females (199/220, 90.5%), while no difference was identified for the right CMA (chi-squared = 0.53; p = 0.47).

## Discussion

Study overview

In the current angiographic study, we studied the DACA variants in a sample of the Greek population. Overall, we identified the typical DACA pattern in the majority of the patients (81.8%), while the bihemispheric, median, and azygos patterns were present in 14.5%, 2.2.%, and 1.5% of the participants, respectively. The present study is, to the best of our knowledge, the first to identify a significant difference in gender-based presence and lateralization of the CMA. In our sample, the CMA was more commonly present in the left hemispheres of males than in those of females, while no gender-based difference in the presence of the CMA was identified in the right hemispheres. This significant gender-based difference in the presence of the CMA in the left hemisphere was also found when the analysis was restricted to patients with a typical DACA pattern.

Typical ACA morphology

In our study, the typical DACA morphology was identified in 81.8% of our sample, while 28.2% presented with a variant pattern. The typical DACA pattern was present in 83.33% of females and in 79.7% of males. The typical anatomy rates in the literature range between 45.2% and 88.6% [10,11,16,17]. Interestingly, Krzyzewski et al. identified a typical pattern with a male predominance (males, 59.7%; females, 46.05%) [11]. In contrast, López-Sala et al. [17] did not identify a statistically significant difference between genders (females, 20.2%; males, 25.4%), similar to Kapoor et al. (females, 52.4%; males, 42.8%) [16].

Distal ACA anomalies

It is important to mention that the only available previous study in a sample of the Greek population [18] did not evaluate the presence or absence of the CMA and did not report on the presence of the bihemispheric type of ACA. The rates of the median (reported as "trifurcation") and azygos (reported as "unpaired") ACA types were 1.5% and 0.4%, respectively [18]. These are very close to those found in our study.

When a median ACA is present, a third DACA usually arises from the anterior communicating artery [19]. In our study, the occurrence of the median ACA type aligns with percentages reported in large (>200 patients) imaging studies from various countries, employing CT angiography (CTA) or MR angiography (MRA), ranging between 0.97% and 5.2% [10,11,17,20-23], except for one study reporting a higher rate of 13.11% [10]. In a previous study, Ogawa et al. utilized a combination of intraoperative observations and DSA to examine anterior circulation variants [10]. They reported a higher prevalence compared to another intraoperative and DSA study [23], attributing it to the interhemispheric surgical technique for approaching the ACA, allowing for better visualization of the anterior circle of Willis. To explain the aforementioned difference, they indicated that in their study, the median ACA was not identifiable on angiography images in 18.52% (5/27) of cases [10]. Indeed, their reported rates more closely resemble that of some earlier cadaveric studies, with median ACA percentages of 8.7% [24] and 13.1% [25]. However, the reported rates in a large cadaveric study (1000 specimens) on the variations of the circle of Willis in India by Kapoor et al. [16] were 2.3%. It is noteworthy that, based on their illustrations, the authors used the term "median ACA" to describe what is otherwise known in the literature as "azygos ACA." Thus, the accurate "median ACA" percentage is reported under the term "triple ACA" [16,19].

The azygos ACA is formed when the two A2 segments are fused and a single vessel provides cerebral irrigation [19]. The rate of azygos ACA in our study aligned with the higher-end rates reported in earlier angiography-based studies, ranging from 0.05% to 1.8% [17,20-22,26,27]. Interestingly and unlike the median ACA type, large cadaveric studies report azygos ACA percentages similar to the angiography-based studies (range: 0.26%-1.7%) [16,25,28].

There have been several anatomical definitions for the bihemispheric ACA pattern [12]. Some authors define it as the one displaying a hypoplastic or early terminating A2 segment with the contralateral A2 providing the major supply of the ACA area in both hemispheres [4,7,19]. In the present study, we used the recent, more inclusive definition proposed by Cilliers and Page [8] indicating that the bihemispheric pattern is the one displaying branches that originate distal to the first cortical ACA branch to supply the contralateral hemisphere, irrespective of the presence of a hypoplastic or early terminating A2 segment [8,12].

Large angiographic studies (>200 patients) have reported a very low rate of the bihemispheric ACA pattern (range: 0.17%-1.8%). However, smaller angiographic studies [29] (15 out of 101 patients, 14.9%) and large cadaveric studies [25] (45 out of 381 brain specimens, 11.8%) have reported percentages closer to our findings. As mentioned by Lehecka et al. [29], modern DSA and CTA techniques, such as those employed in the present study, enable better distinction of vessel aberrations. This is a likely explanation for the higher reported percentage of bihemispheric ACA pattern when compared to other angiographic studies. Nevertheless, given the significant differences in the anatomical definition of the bihemispheric ACA pattern, the apparent variation in reported rates should be interpreted cautiously.

Callosomarginal artery

In the current study, the CMA was present in 90.4% of the studied hemispheres. This finding is consistent with reports from recent studies using similar anatomical definitions for the CMA [9,30]. In a cadaveric study of 100 hemispheres, Ugur et al. reported a CMA rate of 83%, distinguishing between typical (longer course, entering the cingulate sulcus, course parallel to the pericallosal artery) and atypical (shorter course, course not parallel to the pericallosal artery) CMAs [30]. Cavalcanti et al. implemented two different methodologies for studying the CMA morphological anatomy [9]. They used 15 cadaveric brains (30 hemispheres) and supplemented their study with DSA images from 30 hemispheres/sides (20 patients). The overall CMA rate was 93.3% (56 out of 60 hemispheres). Cavalcanti et al. [9] introduced an extension to the classical definition by Moscow et al. [15] for the CMA, the latter defining it as a vessel coursing inside or near the cingulate sulcus, giving rise to at least two cortical branches. In their proposal, Cavalcanti et al. suggest that, when multiple vessels meet the above criteria, the CMA should be designated as the artery with the longer course [9]. The only purely CTA study on the presence of CMA provided a rate of approximately 82% [31].

It is important to note that the rates for the CMA presence vary widely, from as low as 12.4% [32] to as high as 93.4% [33]. After reviewing the existing literature, Table 1 illustrates variations in the applied definitions and reported rates across published studies. This has been previously attributed to the implementation of different definitions by different studies [32,34], highlighting the need for a uniform definition [32].

**Table 1:** Callosomarginal Artery (CMA) in the Literature The table presents the reported prevalence of the callosomarginal artery in various cadaveric and radiologic studies YOP, year of publication; DSA, digital subtraction angiography; CTA, computed tomography angiography; ACA, anterior cerebral artery

First author, YOP	Present CMA/all studied hemispheres (%)	Type of study	CMA definition
Ring and Waddington, 1968 [35]	20/50 cadavers (40%) + 36/100 angiograms (36%)	Cadaveric (50 hemispheres) + angiogram (100 angiograms)	The CMA arises from the pericallosal artery as it runs around the corpus callosum and courses for a considerable distance in the cingulate sulcus to terminate in the paracentral lobule. It seems preferable to confine the term CMA to arteries that follow this course completely
Moscow et al., 1974 [15]	28/54 (58%)	Cadaveric	A vessel coursing in or near the cingulate sulcus, giving rise to two or more cortical branches (Moscow et al. [15])
Bogdanović et al., 1978 [36]	66/86 (76.5%)	Cadaveric	Full text not found (data on prevalence from Lemos [37])
Perlmutter and Rhoton, 1978 [4]	41/50 (82%)	Cadaveric	A vessel coursing in or near the cingulate sulcus, giving rise to two or more cortical branches (Moscow et al. [15])
Lemos, 1984 [37]	34/104 (32.7%)	Cadaveric	A vessel coursing in or near the cingulate sulcus, giving rise to two or more cortical branches (Moscow et al. [15])
Kakou et al., 2000 [38]	39/46 (85%)	Cadaveric	Not mentioned
Ugur et al., 2006 [30]	83/100 (83%), 49 typical and 34 atypical	Cadaveric	Typical (longer course, entering the cingulate sulcus, that runs parallel to the pericallosal), atypical (the lack of a long course where the arteries entered the cingulate sulcus), and absent (the absence of a marked artery within the cingulate sulcus)
Saidi et al., 2008 [34]	66/72 (92%)	Cadaveric	An artery originating from the distal ACA, coursing in the cingulate sulcus, and producing cortical branches
Cavalcanti et al., 2010 [9]	56/60 (93.3%)	Cadaveric (15 brains and 30 hemispheres) + DSA (20 patients and 30 hemispheres)	A vessel coursing in or near the cingulate sulcus, giving rise to two or more cortical branches (Moscow et al. [15]), and, when two or more vessels matched the definition, the one with the longer course within or near the cingulate sulcus was considered as the CMA
Cilliers and Page, 2017 [32]	15/121 (12.4%)	Cadaveric	Typical CMA was defined as an artery running in the cingulate sulcus giving rise to at least two cortical branches (Moscow et al. [15]). The CMA should give at least one artery other than the frontal arteries. If an artery does not run in the cingulate sulcus and gives rise to frontal arteries and other arteries, it should be referred to as an internal frontal artery
Kedia et al., 2013 [33]	28/30 (93.4%), seven typical and 21 atypical	Cadaveric	All CMAs gave at least two branches. Typical (longer course, entering the cingulate sulcus, running parallel to the pericallosal artery with no significant differences between the diameters of the two) and absent (all cortical branches have similar diameter and arose from the pericallosal artery at a right angle, at almost identical distance and directly reached the cortex)
Gomes et al., 1986 [39]	55/60 (91.6%)	Cadaveric	Not mentioned
Kawashima et al., 2003 [40]	20/22 (91%)	Cadaveric	A pericallosal artery branch that courses in or near the cingulate sulcus and gives rise to two or more major cortical branches (Moscow et al. [15])
Mincă et al., 2022 [31]	74/90 (82.2%)	CTA	Several variants of CMA were found: type "0," absent CMA; type "1," CMA with frontoparietal distribution; type "2," CMA with parietal distribution; and type "3," low origin of the CMA, either from the A1 ACA (subtype 3a) or from the initial part of the A2 ACA (subtype 3b). When the CMA continued as pericallosal artery, the CMA was recorded as type "4"
Stefani et al., 2000 [41]	46/78 (59%)	Cadaveric	Major trunk originating from the ACA that runs parallel to it and may be the origin of cortical branches. It has a superior course over the cingulate gyrus or sulcus up to the posterior edge of the paracentral lobule and in close relation with the superior part of the quadrilateral lobule

Cilliers and Page reported that they identified a CMA in only 15 of 121 (12.4%) hemispheres studied [32]. The authors indicate that the distinction between the CMA, as defined by Moscow et al. [15], and the internal frontal artery (an artery following the cingulate sulcus and giving rise to only frontal arteries) has not been made clear in the pertinent literature. They argue that the definition of typical CMA should be altered to specify that an artery should not be named callosomarginal if only frontal branches arise from it. Finally, they suggest that if an artery fits the definition for callosomarginal in regard to its branches but does not course in the cingulate sulcus, it should again be termed an internal frontal artery [32].

Gender-based analysis

Gender differences in morphometric and morphological features of the circle of Willis arteries have been previously established [42-44]. A recent systematic review and meta-analysis of 14 autopsy and radiologic studies highlighted significant variation in the anterior and posterior circulations between males and females [42]. For example, the authors reported that the variants with "one hypoplastic or absent ACA" and "bilateral hypoplastic or absent posterior communicating artery" were more commonly present in males than in females [42]. Furthermore, another systematic review identified gender differences in the length and diameter of ACA, with females having longer and males having wider ACAs [45].

Our findings on the CMA showed a trend for a more common bilateral presence of the vessel in males than in females. Moreover, when analysis was carried out for the two hemispheres separately, a statistically significant difference was found for the left hemisphere where the presence of the CMA was more common in males than females. This gender difference in the rate of CMA is difficult to explain. Regarding the CMA, Lemos [37] cites the evolutionary theory that the emergence of this branch of the ACA is related to the need to irrigate an increasingly large and complex human cortex. Based on this theory, the trend for the more frequent presence of the CMA in males is compatible with the meta-analysis findings of a tendency for larger absolute brain volumes (not body size corrected) in male brains when compared to female brains [46]. Furthermore, the more frequent presence of the CMA in the left hemispheres of males appears consistent with the findings of larger grey matter volumes in the structures of the left hemisphere that are supplied by the CMA and its branches, such as the cingulate gyrus, the precentral gyrus, and the precuneus [46]. While this is a plausible explanation, further radiologic and cadaveric studies should be performed to confirm our findings and to study gender differences in the lateralization of the CMA in different population samples.

Limitations

While our study provides valuable insights regarding the variations of the ACA in a large sample of the Greek population, it has some limitations that should be mentioned. Given the single-center design of the study, it is difficult to draw generalizable conclusions as some regional and demographic variations might have not been captured. Furthermore, retrospective studies have inherent data availability and patient selection biases. In an effort to minimize those, we strictly followed the inclusion criteria and included consecutive patients with complete medical files.

## Conclusions

A better understanding of the anatomy of the ACA and its variation is a prerequisite for accurate clinical interpretations and surgical interventions involving the anterior cerebral circulation. The present angiographic study provided an in-depth look into the DACA variants in a Greek population sample. Moreover, we indicate a significant gender difference in the presence and lateralization of the CMA. Further studies are warranted to confirm our findings and provide further insights into the biological basis underlying this phenomenon.
